# Non-uptake of HIV testing among trans men and trans women: cross-sectional study of client records from 2017 to 2019 in a community-based transgender health center in Metro Manila, Philippines

**DOI:** 10.1186/s12889-022-14158-w

**Published:** 2022-09-16

**Authors:** Zypher Jude G. Regencia, Aisia V. Castelo, Patrick C. Eustaquio, Yanyan S. Araña, John Oliver L. Corciega, John Danvic T. Rosadiño, Ronivin G. Pagtakhan, Emmanuel S. Baja

**Affiliations:** 1 Department of Clinical Epidemiology, College of Medicine, University of the Philippines Manila, City of Manila, 1000 Philippines; 2 Institute of Clinical Epidemiology, National Institutes of Health, University of the Philippines Manila, City of Manila, 1000 Philippines; 3 LoveYourself Inc, Mandaluyong City, 1552 Metro Manila Philippines; 4 Faculty of Management and Development Studies, University of the Philippines - Open University, Los Baños, 4031 Laguna Philippines

**Keywords:** Community-based health center, Gender identity, Non-uptake of HIV testing and counseling (HTC) services, Philippines, Trans men (TM), Trans women (TW)

## Abstract

**Background:**

Transgender individuals are considered at high risk of contracting HIV infection. Integrating HIV testing and counseling (HTC) services into current transgender health programs is necessary to increase its uptake. Our study aimed to describe the characteristics of trans men (TM) and trans women (TW) who accessed HTC services in a community-based transgender health center in Metro Manila, Philippines, and to examine the relationship between gender identity and their non-uptake of HIV testing.

**Methods:**

We conducted a cross-sectional study of TM and TW seeking care from 2017 to 2019. Medical records of clients were reviewed to ascertain their age, gender identity, year and frequency of clinic visits, lifestyle factors, and non-uptake of HIV testing. The effect of gender identity on the non-uptake of HIV testing was estimated using a generalized linear model with Poisson distribution, log link function, and a robust variance, adjusted for confounding variables.

**Results:**

Five hundred twenty-five clients were included in the study, of which about 82.3% (432/525) of the clients declined the HTC services being offered. In addition, the prevalence of non-uptake of HIV testing was 48% higher (Adjusted Prevalence Ratio: 1.48; 95% Confidence Interval: 1.31–1.67) among TM compared to TW. Approximately 3.7% (1/27) and 10.6% (7/66) of the TM and TW, respectively, who accessed the HTC services were reactive. Moreover, most reactive clients were on treatment 87.5% (7/8); three were already virally suppressed, four were on ART but not yet virally suppressed, and one TW client was lost to follow up.

**Conclusion:**

The non-uptake of HTC service of TM and TW is high. HIV program implementers should strategize solutions to reach this vulnerable population for increased and better HTC service uptake and linkage to care.

## Background

The transgender population is recognized as an at-risk group for HIV and other sexually transmitted infections (STI) [1]. Across the world, a pooled HIV prevalence of 19.1% was reported for trans women (TW) [2]. Moreover, the United States Centers for Disease Control and Prevention in 2013 reported that in the 3.3 million HIV testing events conducted, the estimates of transgender individuals newly diagnosed with HIV were nearly three times the national average [3].

This increase in HIV/AIDS cases is consistent with multilevel drivers of HIV among the communities, including social stigma and discrimination [4]. Transgender people are reported to have significantly reduced lifetime rates of HIV testing relative to cisgender gay and bisexual men. Conversely, HIV testing rates are likely lower among transgender adolescents [5]. Increased levels of discrimination, such as denial of medical services and harassment in healthcare settings [6, 7] and expected discrimination, have been associated with postponement or delay of medical services among the transgender population [8, 9]. The current state of our society is directly inclining towards its conventional heteronormative behavior [10]. This ideology increases the vulnerabilities of transgender people to HIV/AIDS in the context of their behaviors, attitudes, and risk practices [11]. They are susceptible to disparities in better access to health, including non-availability of transgender health services, care refusal, substance abuse, and poor mental and sexual health outcomes [12]. This observation parallels narrowed options for their healthcare, such as gender affirmation services, preventive health screenings, and mental health interventions [13].

To address the HIV/AIDS burden, local HIV prevention programs that are trans-inclusive are increasing [14]. Strategies include HIV self-testing, Pre-Exposure Prophylaxis (PrEP) and Post Exposure Prophylaxis (PEP), condoms and lube, and other biopsychosocial methods. Engagement in these programs and services will help mitigate the prevalence of HIV, suicide, and violence across the transgender community [15]. However, the Philippines, where resources are in scarcity, regrettably struggles to address the unique set of healthcare needs the transgender community requires. Moreover, the current healthcare system in the country does not necessarily function effectively for the transgender population, specifically for HIV testing and counseling (HTC) services. As a result, inclusive surveillance and data collection methods across the national transgender communities remain a challenge. Integrating the transgender population into the current Philippines HIV/AIDS surveillance system may modify this current state. Hence, it is essential to establish evidence to support the health outcomes of Filipino transgender people that will help inform program development and interventions explicitly targeted at this key population. Our study aimed to describe the characteristics of trans men (TM) and TW who accessed the community-based transgender health center’s HTC services in Metro Manila, Philippines. Moreover, we examined the relationship between gender identity and the non-uptake of HIV testing in the transgender population.

## Methods

### Study setting

Victoria by Love Yourself Inc. (VLY), the Philippines’ first community-based transgender health center, was established in 2016. Located in Pasay City, Metro Manila, Philippines, the health center receives patients inside and outside said urban capital. This initiative came about in response to the needs of the transgender community, particularly on access to comprehensive and quality transgender healthcare services. It is a one-stop shop that provides holistic care that integrates transgender health and sexual health.

The VLY services include free HTC services, HIV treatment care and support, sexually transmitted infections (STIs) consultation and treatment, PrEP, and PEP. In addition, the center also offers gender-affirming services (GAS), such as gender transitioning counseling, pre-gender affirming surgery assessment and consultation, hormone administration, medically supervised gender-affirming hormone treatment, and even a support group for transgender people. VLY enrolls an average of 21 TM and 8 TW monthly for GAS, whereas around 2 TM and 45 TW visit monthly for their HTC services. HIV testing and treatment services are funded by the Department of Health and Philippine Health Insurance Corporation, the national social health insurance. Additionally, the GAS were initially funded by Stop AIDS Now Fund and LGBT Fund but are now self-sustained by LoveYourself, Inc.

### Study design and population

A cross-sectional study of TM and TW clients seeking care at VLY Community Center from March 2017 to December 2019 was conducted using previously collected clinical/medical records data. We determined their issues relating to their sexual health, particularly their non-uptake of HIV testing. All client records of TM and TW who accessed the services of VLY were screened and included in the study using the following criteria: (1) 18-60 years old and (2) those who identify as transgender, and (3) not those who identify as otherwise including but not limited to cisgender, questioning, or genderqueer/non-binary. Those medical records whose patients’ characteristics fit our criteria were included in our study population.

### Data collection

Medical charts were reviewed to ascertain information from the eligible study participants, including their age, gender identity, initial year and frequency of clinic visits, smoking and drinking statuses, use of recreational drugs, and non-uptake of offered HTC services. Moreover, data extraction of medical records was carried out following a developed case report form. Encoders were trained and ensured to have sufficient expertise, particularly in handling medical records. To identify inaccuracies and discrepancies during the encoding, a small subsample of at least 10% of the total records was reassessed to validate the data encoded into the developed database.

### Exposure and outcome measurements

Gender identity (TW or TM) was the primary exposure in our study. This main exposure was ascertained from the gender identification section of the clients’ medical records. In addition, non-uptake of HIV testing was the outcome of interest among the eligible participants. Non-uptake of HIV testing will be considered if the clients declined or refused HIV testing, while uptake for those who accepted or consented to HIV testing.

### Statistical analysis

Descriptive statistics for the clients’ demographic profile, gender identity, and non-uptake of HIV testing outcome were calculated. The association between gender identity and the non-uptake of HIV testing of the client (refused or declined HIV testing; consented or accepted HIV testing) was estimated using multivariable generalized linear models (GLMs) with a Poisson distribution, log link function, and a robust variance; a suitable method for cross-sectional data with a common outcome [16–18].

**Fig. 1:**
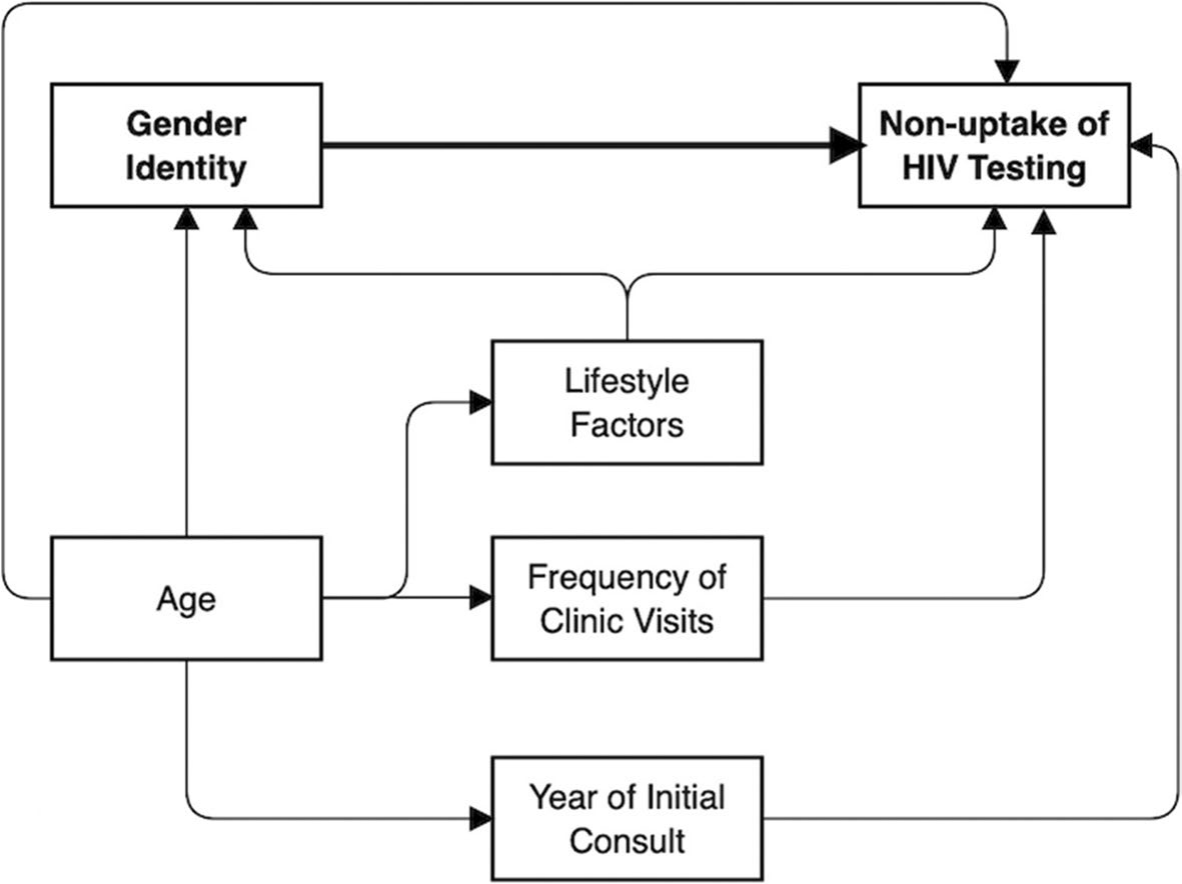
Relationships between the confounding factors, predictors, exposure, and outcome. *Lifestyle factors include drinking status, recreational drug use, and smoking status

In the Poisson model, the following variables were controlled based on previous literature: age (15 – 24 years old; 25 – 34 years old; 35 years old and above), frequency of clinic visit (1 visit; 2 to 3 visits; 4 visits and above), drinking status (never drinker; ever drinker), recreational drug use (never user; ever user), smoking status (never smoker; ever smoker), and year of initial consult (2017; 2018; 2019). The clients’ characteristics included in the model were chosen a priori as potentially important confounding factors and predictors of HIV testing non-uptake (see Fig. 1 for details) [19–32].

For clients who availed of the HTC services of VLY, descriptive statistics were also calculated and stratified by their HIV test results (reactive vs. non-reactive) to summarize the client’s study characteristics.

Sensitivity analysis for unmeasured confounding was estimated using an *E*-value described in the previous report [33]. An adjusted prevalence ratio (aPR) with a 95% confidence interval (95% CI) was used to report the effect size estimate for the effect of gender identity on non-uptake of HIV testing. STATA 17 software (www.stata.com/stata17/) was used to carry out all statistical analyses.

### Ethical approval and consent to participate

Following the national guidelines, the study’s research protocol received ethical approval from the University of the Philippines Manila Research Ethics Board (UPMREB) (CODE: 2021–105-01). The data gathered and client information were kept confidential and private following the Philippine Data Privacy Act of 2012. Written informed consent form from the participants was not required in our study. The need for informed consent was waived by the UPMREB.

## Results

Table 1 shows the descriptive statistics of the characteristics of the study population stratified according to gender identity. A total of 525 TW and TM were included in the study. The clients have a mean age (± SD) of 25.8 ± 5.8 years old. Most of them belonged to the 15–24 years old age bracket (46.7%) and 25–34 years old age bracket (46.1%). Approximately 65.6% of the clients were identified as TM, while the rest were TW. The year 2019, as the initial consult, recorded the highest number of clients (55.6%), which is composed mainly of TM (72.9%), while only 9.9% were recorded as an initial consult during the first year (2017) of the VLY. Regarding the non-uptake of HTC services, approximately 82.3% of the clients refused or declined HIV testing and were mostly TM. Conversely, among the 93 patients who consented to or accepted HIV testing, 27 of them were TM (29.0%) (for details, see Table 1).

**Table 1:** Population study characteristics (*N* = 525)

**Characteristics**^a^	**Total (*N* = 525)**	**Trans Men (*n* = 339)**	**Trans Women (*n* = 186)**
Age, years [mean (standard deviation)]	25.8 (5.8)	26.1 (5.7)	25.4 (6.1)
Age category			
15 – 24 years old	245 (46.7)	153 (45.1)	92 (49.5)
25 – 34 years old	242 (46.1)	162 (47.8)	80 (43.0)
35 years old & above	38 (7.2)	24 (7.1)	14 (7.5)
Year of initial consult			
2017	52 (9.9)	13 (3.8)	39 (21.0)
2018	181 (34.5)	113 (33.3)	68 (36.5)
2019	292 (55.6)	213 (62.9)	79 (42.5)
Total visits			
1 visit	288 (54.9)	166 (49.0)	122 (65.6)
2–3 visits	149 (28.4)	103 (30.4)	46 (24.7)
4 visits & above	88 (16.7)	70 (20.6)	18 (9.7)
Recreational drug			
Ever user	52 (9.9)	39 (11.5)	13 (7.0)
Never user	473 (90.1)	300 (88.5)	173 (93.0)
Smoking status			
Ever smoker	198 (37.7)	148 (43.7)	50 (26.9)
Never smoker	298 (56.8)	174 (51.3)	124 (66.7)
Missing data	29 (5.5)	17 (5.0)	12 (6.4)
Drinking status			
Ever drinker	430 (81.9)	294 (86.7)	136 (73.1)
Never drinker	70 (13.3)	31 (9.2)	39 (21.0)
Missing data	25 (4.8)	14 (4.1)	11 (5.9)
Non-uptake status			
Consented/Accepted HIV testing	93 (17.7)	27 (8.0)	66 (35.5)
Refused/Declined HIV testing	432 (82.3)	312 (92.0)	120 (64.5)

^a^Distributions of variables are reported as n (%) unless otherwise specified

Table 2 shows the adjusted effect estimate of gender identity on non-uptake of HIV testing. The prevalence of not getting tested for HIV is 48.0% higher among TM clients (aPR: 1.48; 95% CI: 1.31 to 1.67; *p*-value < 0.001) compared to TW clients. Table 2 also shows the *E*-values for the point estimate and the confidence interval as a result of the sensitivity analysis of the unmeasured confounding. With an observed point estimate prevalence ratio of 1.48, an unmeasured confounder associated with both gender identity and non-uptake of HIV testing by a prevalence ratio of 2.32 each, above and beyond the measured confounder, could explain away the prevalence ratio estimate. But weaker confounding could not. In addition, with an observed lower bound confidence interval prevalence ratio of 1.31, an unmeasured confounder was associated with both gender identity and non-uptake of HIV testing by a prevalence ratio of 1.95 each, above and beyond the measured confounders, could shift the confidence interval to include the null. However, weaker confounding could not.

**Table 2:** Adjusted prevalence ratio (aPR) with a 95% confidence interval (CI) for the association between gender identity and the non-uptake of HIV testing among TM & TW

**Exposure** ^a^	**Total (*n* = 525)**	**Uptake: Consented/ Accepted HIV Testing (*n* = 93)**	**Non-Uptake Refused/ Declined HIV Testing (***n* = **432)**	**aPR (95% CI) for the non-uptake of HIV testing**	***E*-value for point estimate**	***E*-value for CI**
Gender identity					2.32	1.95
Trans woman	186	66 (71.0%)	120 (27.8%)	1.0		
Trans man	339	27 (29.0%)	312 (72.2%)	1.48 (1.31, 1.67)^b^		

^a^Distribution of gender identity exposure is reported as n (%)

^b^*p*-value < 0.001

Clients who consented to or accepted HTC services from the VLY were further described in Table 3 and stratified according to their HIV test results. Regarding gender identity, 96.3% and 89.4% of the TM and TW clients were non-reactive, respectively, which implied that TW had a higher HIV prevalence rate than TM clients (10.6% vs. 3.7%). Moreover, reactive patients were only observed in 2018 & 2019, with approximately one out of ten transgender people testing reactive for HIV. Furthermore, 2019 recorded the most clients who availed the HTC services, 57.0% (53/93). Out of the eight reactive clients, most were on ART treatment 87.5% (7/8); three were already virally suppressed, four were not yet virally suppressed, and one TW was lost to follow up.

**Table 3:** Characteristics of patients who availed of HIV Testing and Counselling (HTC) services, stratified by their HIV test result (*N* = 93)

**Characteristics**^a^	**Total** (*n* = 93)	**Non-Reactive** (*n* = 85)	**Reactive** (*n* = 8)
Age, years [mean (SD)]	25.6 (5.7)	25.4 (5.6)	27.5 (6.9)
Age category			
15 – 24 years old	45	42 (93.3%)	3 (6.7%)
25 – 34 years old	42	38 (90.5%)	4 (9.5%)
> 35 years old	6	5 (83.3.9%)	1 (16.7%)
Gender identity			
Trans man	27	26 (96.3%)	1 (3.7%)
Trans woman	66	59 (89.4%)	7 (10.6%)
Year of initial consult			
2017	9	9 (100.0%)	0 (0.0%)
2018	31	28 (90.3%)	3 (9.7%)
2019	53	48 (90.6%)	5 (9.4%)
Total visits			
1 visit	56	50 (89.3%)	6 (10.7%)
2–3 visits	29	27 (93.1%)	2 (6.7%)
4 visits & above	8	8 (100.0%)	0 (0.0%)
Recreational drug			
Ever user	11	11 (100.0%)	0 (0.0%)
Never user	82	74 (90.2%)	8 (9.8%)
Smoking status			
Ever smoker	31	31 (100.0%)	0 (0.0%)
Never smoker	58	50 (86.2.8%)	8 (13.8%)
Missing data	4	4 (100.0%)	0 (0.0%)
Drinking status			
Ever drinker	72	66 (91.7%)	6 (8.3%)
Never drinker	17	15 (88.2.6%)	2 (11.8%)
Missing data	4	4 (100.0%)	0 (0.0%)

^a^Distributions of variables are reported as n (%) unless specified otherwise

## Discussion

Several factors may increase the risk of transgender (TG) populations for HIV infection. TW were identified as having more significant risks of acquiring HIV infection than TM. TG populations were also least likely to receive HIV treatments or interventions and other preventative services [2, 34–36]. The TG community is also known to experience an increased risk for sexual behaviors, family rejection, stigma, discrimination, and safety concerns [37–39]. In addition, numerous individual, social, and interpersonal factors provide an interplay in terms of the experiences the TG community endures [40, 41].

A report on the education and training for health professionals in the Philippines provided information on the adequacy of the current health curricula in terms of the HIV response [42]. Moreover, the Integrated HIV Behavioral and Serologic Surveillance embedded in the Health Sector Plan for HIV and STI 2015 to 2020 of the Philippine Department of Health (DOH), an active sentinel serologic and behavioral surveillance, suggested actions to increase HIV and HIV-related services both for the TG and men-having-sex-with-men (MSM) populations [43, 44]. However, the guidelines for the increase in uptake of HTC services among the TW and TM should be further strengthened because of the existing barriers to testing [45]. Our study aimed to identify gender identity as a factor that enables the TG populations to refuse or decline HIV testing services. Through medical records review, our study showed that most TM did not consent or accept HIV testing services from the VLY, and they are more likely to refuse HIV testing services compared to TG.

Our results conformed with the prevalence report of the US CDC recommended guidelines for HIV and STI, wherein suboptimal trends in HTC services were observed among TG [46]. This finding is congruent with the results that TM did not know their HIV status [47]. However, one study on TG youth showed that TW was significantly less likely to get tested for HIV compared with TM [48]. Contrary to this finding, a recent publication on an extensive survey from the United States consisting of 26,927 TG respondents in 2015 revealed that TW had significantly higher odds of reporting their HIV status than TM [49], which was also seen in our study. In addition, the most common reason for never testing for HIV among TM was a low-risk perception of their sexual activities. Low-risk perception as a significant barrier to HIV testing was also seen in previous studies [50–53], not only among TG populations. Other reasons for TM or TW not getting their HIV testing also included fear of HIV-related stigma and discrimination [54, 55], insufficient knowledge on HIV/AIDS or poor health literacy [56, 57], and limited availability due to lack of time [58]. Further investigation on why TM and TW in VLY do not know their HIV status because of refusal should be conducted to engage more TM and TW clients in HTC services. Moreover, increasing the willingness for HIV self-testing among TM or TW to ensure one’s safety and confidentiality is an alternative approach that can also be explored [59, 60].

The third year since the launch of VLY in 2017 recorded the highest number of TM and TW clients consenting to HIV testing. As an exclusive health center for the TG community under the supervision of LoveYourself Inc., VLY was initially established to provide HTC [61]. Over the years, through community consultations, partnerships with LGBTQIA + organizations, and TG health capacity building of the community center, VLY officially rolled out their GAS, the first in the Philippines. The one-stop-shop model of integrating sexual health services and TG health could translate to a gradual increase in HIV testing uptake among VLY clients. This strategy further establishes that gender-affirming care services can be an entry point in accessing HIV services.

Similarly, research has suggested that a gender-affirmative integrated care framework complemented by peer navigation effectively addresses the HIV burden experienced by the TG population [62]. Our results also showed that the later years of VLY operations had more TM and TW clients encouraged to avail themselves of the HTC services. In previous reports, this observation was also seen in other centers/clinics [63, 64]. Building trust and rapport between physicians, HTC service providers, and clients are crucial in all central HIV testing practices [64, 65]. The integral approach to establishing trust is, to begin with, simple steps, taking part in clients with small successes and showing dedication and commitment through continuous communication [66]. VLY used this strategy to build trust and rapport with TW and TM clients, which was also seen in previous studies conducted by other HTC providers [67, 68]. The establishment of VLY as a community-based TG health center for TM and TW provides an essential avenue for these populations to avail of HTC services in confidence and without stigma and discrimination.

HIV testing lacking good motivational counseling and linkage to care may not be effective [69]. Our study provided information on reactive TG clients in which almost all were linked to care, particularly HIV treatment through ART. Engagement in HIV care among all vulnerable populations, not only TG clients, is essential in the HIV care continuum. The role of HTC service providers, such as VLY, in the delivery of services and building relationships is characterized by their provision of time and emotional and social support to their clients [70, 71]. However, previous studies documented low ART coverage among TG respondents [72–74]. Nevertheless, our findings demonstrated improved access and link to care among HIV-reactive TW and TM clients of VLY, similar to another published study [75].

To our knowledge, this is the first quantitative cross-sectional study conducted in the Philippines that looked at the non-uptake of HIV testing of TM and TW clients. Using a modest sample size of client records, we presented the disparity between TM and TW regarding the non-uptake of HTC services in Metro Manila. Through continuous monitoring and engagement of TM and TW in the community-based TG health center environment, the identified disparity provided an opportunity for the VLY to enhance its services for TM and TW accessing HTC services. Furthermore, by gradually eliminating this gap through the retention of commitment and trust built around with clients and proper dissemination of the availability of HTC services for the TM and TW community, HIV cases in Metro Manila may be reduced.

However, the current study is limited by its secondary data analysis nature, in which participant information was collected through a medical chart review. We acknowledged that missing data in our medical chart documentation may have precluded insightful analysis of other potential predictors. Given its cross-sectional study design, it could not identify the temporal and causal relationships between gender identity and the non-uptake of HIV testing. In addition, our study focused on individual-level exposures rather than societal-level exposures. Additionally, gender identity was the main exposure of our study, wherein it was treated as an attribute that may be relevant to the individual’s health [76]. Hence, other study designs are recommended in future studies to establish causality between the exposure and the outcome of interest, such as randomized control trials.

Moreover, possible selection bias from the sample population could not be ignored because the distribution characteristics of our study population who had access to VLY might not have the same distribution characteristics of those who do not have access to VLY services. Furthermore, our study did not account for the sexual orientation of the study population. TM who have sex with cisgender men and TW are increasingly at risk of HIV. Given the current growing number of national and global programs focused on TW, not TG in general, the exploration of HIV testing uptake in terms of sexual orientation has important implications on TM not perceiving they are actually at risk for the HIV infection. In addition, the potential effect of residual unmeasured confounding factor (*i.e.,* HIV-related social stigma and discrimination) bias could not be ruled out. Furthermore, we only involved TM and TW clients who accessed VLY from 2017 to 2019, which may not be representative of other TM and TW clients in other parts of the Philippines, other races, and other vulnerable populations at-risk for HIV. Further studies are needed to validate our findings across different populations and other community centers.

## Conclusion

The role of early HTC services in the reduction of increasing HIV cases is an essential approach in the HIV care spectrum, especially for vulnerable populations such as the TG community. In our study, the non-uptake of HTC service of TM and TW is high. Our study, which demonstrated the refusal rate of HIV testing among TG populations, particularly among TM, presented an opportunity for the HIV program implementers in the Philippines to reach this group to provide the HTC services they need.

## Data Availability

The original data are not available for sharing to protect the clients’ confidentiality. An email may be sent to the corresponding author, Dr. Emmanuel S. Baja (esbaja@up.edu.ph), for further inquiries.

## Abbreviations

aPR: Adjusted Prevalence Ratio
ART: Antiretroviral therapy
CDC: Centers for Disease Control
CI: Confidence Interval
DOH: Department of Health
GLM: Generalized Linear Models
HIV: Human Immunodeficiency Virus
HTC: HIV Testing and Counselling
MSM: Men-having-sex-with-men
PrEP: Pre-Exposure Prophylaxis
PEP: Post-Exposure Prophylaxis
SD: Standard Deviation
STI: Sexually Transmitted Infections
TG: Transgender
TM: Trans men
TW: Trans women
VLY: Victoria by LoveYourself Inc
